# 1,1′-Bicyclo­hexyl-1,1′-diyl 2,2′-bipyridine-3,3′-dicarboxyl­ate

**DOI:** 10.1107/S1600536812018508

**Published:** 2012-05-05

**Authors:** Hoong-Kun Fun, Ming Yeng Lim, Ching Kheng Quah, Dongdong Wu

**Affiliations:** a X-ray Crystallography Unit, School of Physics, Universiti Sains Malaysia, 11800 USM, Penang, Malaysia; b School of Chemistry and Chemical Engineering, Nanjing University, Nanjing 210093, People’s Republic of China

## Abstract

The title compound, C_24_H_26_N_2_O_4_, lies about a crystallographic twofold rotation axis. The cyclo­hexane rings adopts a chair conformation. The two pyridine rings form a dihedral angle of 41.02 (4)°. In the crystal, mol­ecules are linked *via* C—H⋯O and C—H⋯N hydrogen bonds into a layer parallel to the *bc* plane.

## Related literature

For the background to this study, see the first paper in this series: Fun, Quah, Wu & Zhang (2012 ▶). For a related structure, see: Fun, Quah & Wu (2012 ▶). For the stability of the temperature controller used in the the data collection, see: Cosier & Glazer (1986 ▶). For standard bond-length data, see: Allen *et al.* (1987 ▶). For ring conformations, see: Cremer & Pople (1975 ▶). For the preparation, see: Wu *et al.* (2012 ▶).
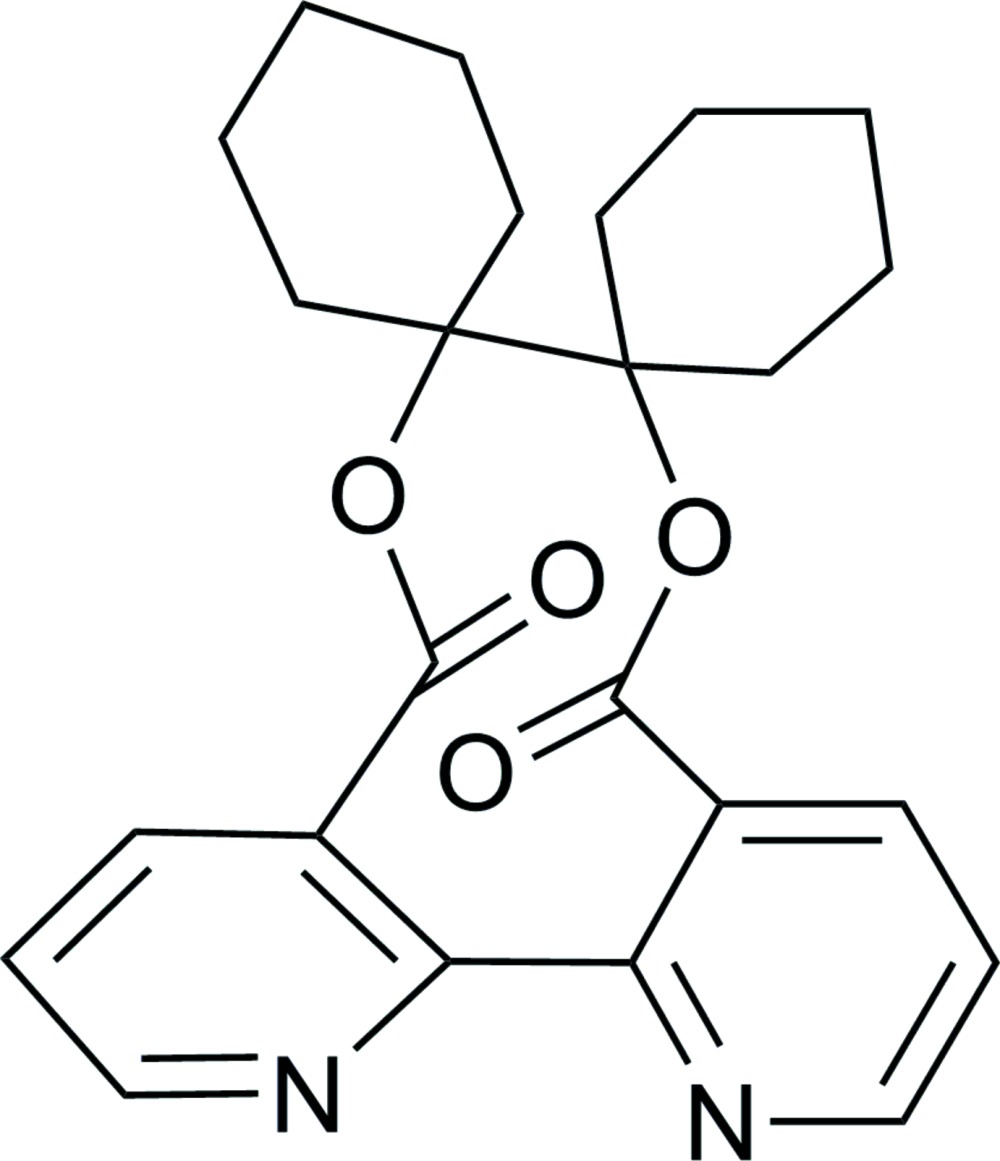


## Experimental

### 

#### Crystal data

C_24_H_26_N_2_O_4_
*M* *_r_* = 406.47
Monoclinic, 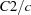

*a* = 16.7647 (3) Å
*b* = 10.2618 (2) Å
*c* = 11.5755 (2) Å
β = 99.810 (1)°
*V* = 1962.28 (6) Å^3^
*Z* = 4
Mo *K*α radiation
μ = 0.09 mm^−1^
*T* = 100 K
0.53 × 0.24 × 0.12 mm

#### Data collection

Bruker SMART APEXII CCD area-detector diffractometer
Absorption correction: multi-scan (*SADABS*; Bruker, 2009 ▶) *T*_min_ = 0.952, *T*_max_ = 0.989
11734 measured reflections
3578 independent reflections
2972 reflections with *I* > 2σ(*I*)
*R*_int_ = 0.027

#### Refinement

*R*[*F* ^2^ > 2σ(*F* ^2^)] = 0.043
*wR*(*F* ^2^) = 0.119
*S* = 1.04
3578 reflections
136 parameters
H-atom parameters constrained
Δρ_max_ = 0.50 e Å^−3^
Δρ_min_ = −0.27 e Å^−3^

### 

Data collection: *APEX2* (Bruker, 2009 ▶); cell refinement: *SAINT* (Bruker, 2009 ▶); data reduction: *SAINT*; program(s) used to solve structure: *SHELXTL* (Sheldrick, 2008 ▶); program(s) used to refine structure: *SHELXTL*; molecular graphics: *SHELXTL*; software used to prepare material for publication: *SHELXTL* and *PLATON* (Spek, 2009 ▶).

## Supplementary Material

Crystal structure: contains datablock(s) global, I. DOI: 10.1107/S1600536812018508/is5124sup1.cif

Structure factors: contains datablock(s) I. DOI: 10.1107/S1600536812018508/is5124Isup2.hkl

Supplementary material file. DOI: 10.1107/S1600536812018508/is5124Isup3.cml

Additional supplementary materials:  crystallographic information; 3D view; checkCIF report

## Figures and Tables

**Table 1:** Hydrogen-bond geometry (Å, °)

*D*—H⋯*A*	*D*—H	H⋯*A*	*D*⋯*A*	*D*—H⋯*A*
C3—H3*A*⋯O2^i^	0.95	2.60	3.5319 (12)	168
C12—H12*A*⋯N1^ii^	0.99	2.54	3.4641 (12)	156

Symmetry codes: (i) 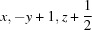; (ii) 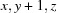.

**Fig. 1.**
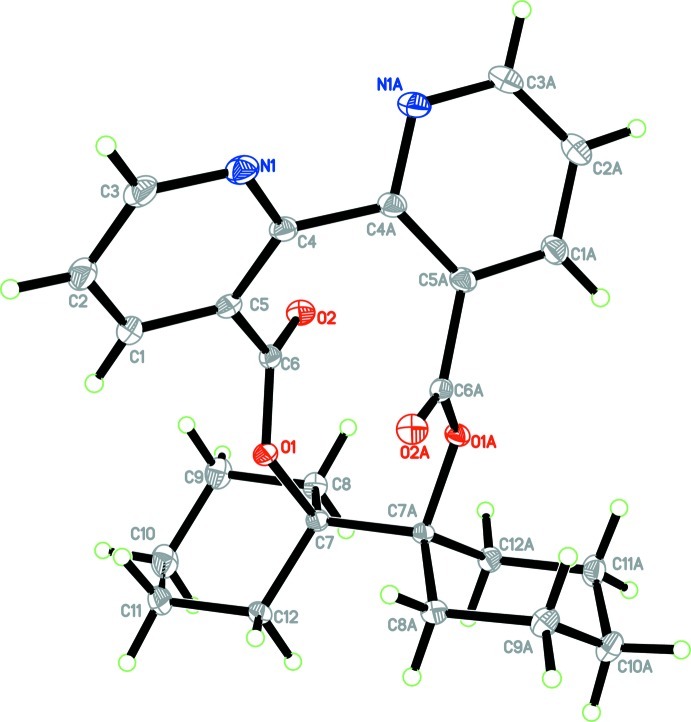
The molecular structure of the title compound, showing 50% probability displacement ellipsoids for non-H atoms. Atoms with suffix A have been generated by the symmetry code -x, y, -z + 1/2.

**Fig. 2.**
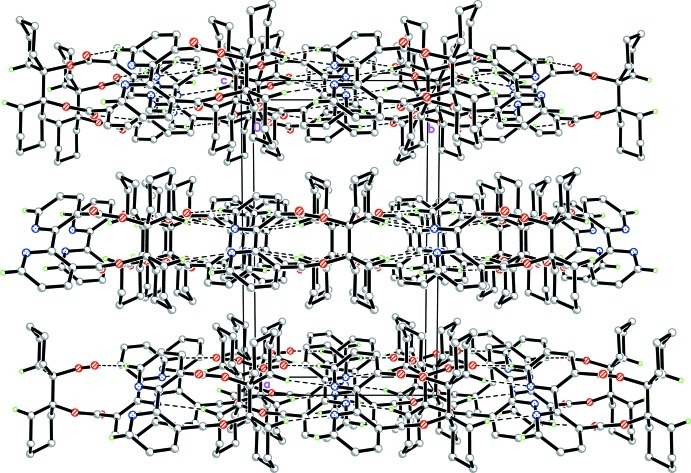
A packing diagram of the title compound, viewed along the c axis. H atoms not involved in hydrogen bonds (dashed lines) have been omitted for clarity.

## supplementary crystallographic information

### Comment

The title compound is a ten-membered bislactone with biaryl moiety fused. Easy preparation of this compound could be achieved through a concise photochemical method (Wu *et al.*, 2012). The title compound, Fig. 1, lies about a crystallographic twofold axis generated by the symmetry code -*x*, *y*, -*z* + 1/2. The cyclohexane ring (C7–C12) adopts a chair conformation with puckering parameters (Cremer & Pople, 1975) Q = 0.5773 (10) Å, Θ = 177.88 (10)° and φ = 256 (3)°. The two pyridine rings (N1/C1–C5 & N1A/C1A–C5A) are essentially planar [maximum deviation of 0.018 (1) Å at atom C4/C4A] and form a dihedral angle of 41.02 (4)°. Bond lengths (Allen *et al.*, 1987) and angles are within normal ranges and are comparable to related structures (Fun, Quah, Wu & Zhang, 2012; Fun, Quah & Wu, 2012). In the crystal (Fig. 2), molecules are linked *via* intermolecular C3—H3A···O2 and C12—H12A···N1 hydrogen bonds (Table 1) into layers parallel to the (100) plane.

### Experimental

The title compound was the product from the photo-oxidation between 2,3-dispirohexyl-2,3-dihydro-[1,4]dioxino[2,3-f][1,10]phenanthroline and oxygen. The compound was purified by flash column chromatography with ethyl acetate/petroleum ether (1:10) as eluents. X-ray quality crystals of the title compound (*m.p.* 180–183 °C), were obtained from slow evaporation of an acetone and petroleum ether solution (1:10).

### Refinement

All H atoms were positioned geometrically and refined using a riding model with C—H = 0.95 or 0.99 Å and *U*_iso_(H) = 1.2*U*_eq_(C).

### Crystal data

**Table:**

C_24_H_26_N_2_O_4_	*F*(000) = 864
*M**_r_* = 406.47	*D*_x_ = 1.376 Mg m^−^^3^
Monoclinic, *C*2/*c*	Mo *K*α radiation, λ = 0.71073 Å
Hall symbol: -C 2yc	Cell parameters from 4821 reflections
*a* = 16.7647 (3) Å	θ = 2.3–32.4°
*b* = 10.2618 (2) Å	µ = 0.09 mm^−^^1^
*c* = 11.5755 (2) Å	*T* = 100 K
β = 99.810 (1)°	Block, colourless
*V* = 1962.28 (6) Å^3^	0.53 × 0.24 × 0.12 mm
*Z* = 4	

### Data collection

**Table:**

Bruker SMART APEXII CCD area-detector diffractometer	3578 independent reflections
Radiation source: fine-focus sealed tube	2972 reflections with *I* > 2σ(*I*)
Graphite monochromator	*R*_int_ = 0.027
φ and ω scans	θ_max_ = 32.7°, θ_min_ = 2.3°
Absorption correction: multi-scan (*SADABS*; Bruker, 2009)	*h* = −24→25
*T*_min_ = 0.952, *T*_max_ = 0.989	*k* = −15→15
11734 measured reflections	*l* = −14→17

### Refinement

**Table:**

Refinement on *F*^2^	Primary atom site location: structure-invariant direct methods
Least-squares matrix: full	Secondary atom site location: difference Fourier map
*R*[*F*^2^ > 2σ(*F*^2^)] = 0.043	Hydrogen site location: inferred from neighbouring sites
*wR*(*F*^2^) = 0.119	H-atom parameters constrained
*S* = 1.04	*w* = 1/[σ^2^(*F*_o_^2^) + (0.0602*P*)^2^ + 1.2358*P*] where *P* = (*F*_o_^2^ + 2*F*_c_^2^)/3
3578 reflections	(Δ/σ)_max_ = 0.001
136 parameters	Δρ_max_ = 0.50 e Å^−^^3^
0 restraints	Δρ_min_ = −0.27 e Å^−^^3^

### Special details

**Table:**

Experimental. The crystal was placed in the cold stream of an Oxford Cryosystems Cobra open-flow nitrogen cryostat (Cosier & Glazer, 1986) operating at 100.0 (1) K.
Geometry. All esds (except the esd in the dihedral angle between two l.s. planes) are estimated using the full covariance matrix. The cell esds are taken into account individually in the estimation of esds in distances, angles and torsion angles; correlations between esds in cell parameters are only used when they are defined by crystal symmetry. An approximate (isotropic) treatment of cell esds is used for estimating esds involving l.s. planes.
Refinement. Refinement of F^2^ against ALL reflections. The weighted R-factor wR and goodness of fit S are based on F^2^, conventional R-factors R are based on F, with F set to zero for negative F^2^. The threshold expression of F^2^ > 2sigma(F^2^) is used only for calculating R-factors(gt) etc. and is not relevant to the choice of reflections for refinement. R-factors based on F^2^ are statistically about twice as large as those based on F, and R- factors based on ALL data will be even larger.

### Fractional atomic coordinates and isotropic or equivalent isotropic displacement parameters (Å^2^)

**Table:**

	*x*	*y*	*z*	*U*_iso_*/*U*_eq_	
O1	0.07035 (4)	0.91569 (6)	0.32195 (5)	0.01138 (14)	
O2	0.08919 (4)	0.79994 (7)	0.15933 (6)	0.01480 (15)	
N1	0.03750 (5)	0.47163 (8)	0.36731 (7)	0.01641 (17)	
C1	0.13527 (6)	0.68507 (10)	0.44475 (8)	0.01575 (18)	
H1A	0.1678	0.7583	0.4717	0.019*	
C2	0.13993 (6)	0.57190 (10)	0.51086 (8)	0.01792 (19)	
H2A	0.1757	0.5658	0.5837	0.022*	
C3	0.09090 (6)	0.46750 (10)	0.46771 (8)	0.01813 (19)	
H3A	0.0955	0.3889	0.5117	0.022*	
C4	0.03211 (5)	0.58187 (9)	0.30384 (8)	0.01315 (17)	
C5	0.08212 (5)	0.68998 (9)	0.33802 (8)	0.01229 (16)	
C6	0.08110 (5)	0.80731 (9)	0.26097 (8)	0.01163 (16)	
C7	0.04781 (5)	1.04426 (9)	0.26757 (7)	0.01063 (16)	
C8	0.09239 (5)	1.07527 (9)	0.16519 (8)	0.01365 (17)	
H8A	0.0760	1.0116	0.1013	0.016*	
H8B	0.0764	1.1631	0.1343	0.016*	
C9	0.18467 (6)	1.07060 (10)	0.20256 (9)	0.01769 (19)	
H9A	0.2013	0.9814	0.2289	0.021*	
H9B	0.2110	1.0921	0.1346	0.021*	
C10	0.21245 (6)	1.16711 (11)	0.30191 (9)	0.0207 (2)	
H10A	0.2716	1.1591	0.3280	0.025*	
H10B	0.2007	1.2572	0.2734	0.025*	
C11	0.16867 (6)	1.13963 (10)	0.40464 (8)	0.01758 (19)	
H11A	0.1842	1.2065	0.4660	0.021*	
H11B	0.1860	1.0537	0.4388	0.021*	
C12	0.07650 (5)	1.14012 (9)	0.36753 (8)	0.01346 (17)	
H12A	0.0585	1.2291	0.3422	0.016*	
H12B	0.0510	1.1171	0.4359	0.016*	

### Atomic displacement parameters (Å^2^)

**Table:**

	*U*^11^	*U*^22^	*U*^33^	*U*^12^	*U*^13^	*U*^23^
O1	0.0130 (3)	0.0087 (3)	0.0120 (3)	0.0012 (2)	0.0009 (2)	0.0006 (2)
O2	0.0162 (3)	0.0141 (3)	0.0145 (3)	0.0007 (2)	0.0040 (2)	−0.0013 (2)
N1	0.0203 (4)	0.0111 (4)	0.0178 (3)	0.0014 (3)	0.0030 (3)	0.0021 (3)
C1	0.0156 (4)	0.0141 (4)	0.0164 (4)	0.0025 (3)	−0.0004 (3)	−0.0002 (3)
C2	0.0196 (4)	0.0177 (5)	0.0154 (4)	0.0054 (4)	−0.0002 (3)	0.0016 (3)
C3	0.0223 (4)	0.0140 (4)	0.0180 (4)	0.0052 (4)	0.0032 (3)	0.0039 (3)
C4	0.0156 (4)	0.0099 (4)	0.0138 (4)	0.0012 (3)	0.0022 (3)	0.0001 (3)
C5	0.0132 (3)	0.0097 (4)	0.0139 (3)	0.0020 (3)	0.0020 (3)	0.0005 (3)
C6	0.0094 (3)	0.0098 (4)	0.0151 (4)	−0.0001 (3)	0.0003 (3)	−0.0002 (3)
C7	0.0118 (3)	0.0076 (3)	0.0119 (3)	−0.0002 (3)	0.0003 (3)	0.0009 (3)
C8	0.0132 (4)	0.0137 (4)	0.0140 (4)	−0.0011 (3)	0.0023 (3)	0.0021 (3)
C9	0.0134 (4)	0.0198 (5)	0.0203 (4)	−0.0020 (3)	0.0040 (3)	0.0032 (4)
C10	0.0143 (4)	0.0193 (5)	0.0271 (5)	−0.0050 (4)	−0.0001 (3)	0.0018 (4)
C11	0.0141 (4)	0.0167 (4)	0.0200 (4)	−0.0014 (3)	−0.0028 (3)	−0.0025 (4)
C12	0.0142 (4)	0.0101 (4)	0.0148 (4)	−0.0005 (3)	−0.0011 (3)	−0.0023 (3)

### Geometric parameters (Å, º)

**Table:**

O1—C6	1.3458 (11)	C7—C7^i^	1.5854 (17)
O1—C7	1.4828 (11)	C8—C9	1.5344 (13)
O2—C6	1.2094 (11)	C8—H8A	0.9900
N1—C3	1.3414 (12)	C8—H8B	0.9900
N1—C4	1.3435 (12)	C9—C10	1.5290 (15)
C1—C2	1.3856 (14)	C9—H9A	0.9900
C1—C5	1.3956 (12)	C9—H9B	0.9900
C1—H1A	0.9500	C10—C11	1.5264 (15)
C2—C3	1.3903 (15)	C10—H10A	0.9900
C2—H2A	0.9500	C10—H10B	0.9900
C3—H3A	0.9500	C11—C12	1.5317 (13)
C4—C5	1.4061 (13)	C11—H11A	0.9900
C4—C4^i^	1.5018 (18)	C11—H11B	0.9900
C5—C6	1.4968 (12)	C12—H12A	0.9900
C7—C12	1.5324 (12)	C12—H12B	0.9900
C7—C8	1.5382 (12)		
			
C6—O1—C7	124.05 (6)	C7—C8—H8A	109.2
C3—N1—C4	118.24 (9)	C9—C8—H8B	109.2
C2—C1—C5	119.12 (9)	C7—C8—H8B	109.2
C2—C1—H1A	120.4	H8A—C8—H8B	107.9
C5—C1—H1A	120.4	C10—C9—C8	110.80 (8)
C1—C2—C3	118.24 (9)	C10—C9—H9A	109.5
C1—C2—H2A	120.9	C8—C9—H9A	109.5
C3—C2—H2A	120.9	C10—C9—H9B	109.5
N1—C3—C2	123.60 (9)	C8—C9—H9B	109.5
N1—C3—H3A	118.2	H9A—C9—H9B	108.1
C2—C3—H3A	118.2	C11—C10—C9	109.94 (8)
N1—C4—C5	121.94 (8)	C11—C10—H10A	109.7
N1—C4—C4^i^	115.20 (6)	C9—C10—H10A	109.7
C5—C4—C4^i^	122.84 (6)	C11—C10—H10B	109.7
C1—C5—C4	118.74 (8)	C9—C10—H10B	109.7
C1—C5—C6	119.82 (8)	H10A—C10—H10B	108.2
C4—C5—C6	121.42 (8)	C10—C11—C12	112.15 (8)
O2—C6—O1	127.53 (8)	C10—C11—H11A	109.2
O2—C6—C5	122.52 (8)	C12—C11—H11A	109.2
O1—C6—C5	109.95 (7)	C10—C11—H11B	109.2
O1—C7—C12	103.07 (6)	C12—C11—H11B	109.2
O1—C7—C8	112.90 (7)	H11A—C11—H11B	107.9
C12—C7—C8	108.53 (7)	C11—C12—C7	112.42 (8)
O1—C7—C7^i^	106.40 (5)	C11—C12—H12A	109.1
C12—C7—C7^i^	111.54 (7)	C7—C12—H12A	109.1
C8—C7—C7^i^	113.91 (8)	C11—C12—H12B	109.1
C9—C8—C7	112.04 (7)	C7—C12—H12B	109.1
C9—C8—H8A	109.2	H12A—C12—H12B	107.9
			
C5—C1—C2—C3	−0.15 (14)	C1—C5—C6—O1	−53.79 (11)
C4—N1—C3—C2	1.26 (15)	C4—C5—C6—O1	128.11 (9)
C1—C2—C3—N1	−2.13 (16)	C6—O1—C7—C12	−157.78 (7)
C3—N1—C4—C5	1.90 (14)	C6—O1—C7—C8	−40.90 (10)
C3—N1—C4—C4^i^	−176.74 (10)	C6—O1—C7—C7^i^	84.75 (10)
C2—C1—C5—C4	3.07 (14)	O1—C7—C8—C9	−56.99 (10)
C2—C1—C5—C6	−175.08 (8)	C12—C7—C8—C9	56.61 (10)
N1—C4—C5—C1	−4.06 (14)	C7^i^—C7—C8—C9	−178.48 (6)
C4^i^—C4—C5—C1	174.47 (10)	C7—C8—C9—C10	−58.49 (11)
N1—C4—C5—C6	174.05 (8)	C8—C9—C10—C11	56.01 (11)
C4^i^—C4—C5—C6	−7.41 (15)	C9—C10—C11—C12	−54.88 (11)
C7—O1—C6—O2	14.66 (13)	C10—C11—C12—C7	55.67 (11)
C7—O1—C6—C5	−165.46 (7)	O1—C7—C12—C11	65.03 (9)
C1—C5—C6—O2	126.10 (10)	C8—C7—C12—C11	−54.90 (10)
C4—C5—C6—O2	−52.00 (13)	C7^i^—C7—C12—C11	178.81 (7)

Symmetry code: (i) −*x*, *y*, −*z*+1/2.

### Hydrogen-bond geometry (Å, º)

**Table:**

*D*—H···*A*	*D*—H	H···*A*	*D*···*A*	*D*—H···*A*
C3—H3*A*···O2^ii^	0.95	2.60	3.5319 (12)	168
C12—H12*A*···N1^iii^	0.99	2.54	3.4641 (12)	156

Symmetry codes: (ii) *x*, −*y*+1, *z*+1/2; (iii) *x*, *y*+1, *z*.
